# Migration of liver sinusoidal leukocytes to the liver colon adenocarcinoma metastases

**DOI:** 10.1186/1476-5926-2-S1-S53

**Published:** 2004-01-14

**Authors:** Sergiusz Durowicz, Barbara Lukomska, Joanna Dłułniewska, Dorota Laszuk, Waldemar L Olszewski

**Affiliations:** 1 Medical Research Center, Polish Academy of Sciences Department of Surgical Research & Transplantology, 02-106 Warsaw, Poland

## Introduction

Liver is an organ with three populations of host cells reacting with tumor cells. These are the Kupffer cells and macrophages [1,2], sinusoidal NK cells [3,4] and cells mobilized from the portal blood upon chemotactic stimuli [5]. There is a large literature on the role of liver sedentary macrophages in tumor cell elimination [6]. On the other hand, little is known about the in vivo contact in sinusoids between population of temporarily halted leukocytes and the colon metastatic tumor cells.

The following questions were asked: (a) which blood leukocyte populations are transiently halted in sinusoids of livers with adenocarcinoma metastases, compared with normal livers, (b) which adhesion molecules play role in blood immune cells trapping in the metastatic foci, and (c) what are the phenotypical similarities between the sinusoidal blood populations washed-out from livers with cancer and cellular infiltrates in and around the metastatic foci.

## Methods

Wistar (W/Wag) rats weighing 200ł300 g were used. Liver tumors were induced by injection of 1 ł 10^6 ^of CC531 colon cancer cells into the portal vein. Rats were anesthetized and heparinized. For liver perfusion portal vein and suprahepatic vena cava were cannulated. The infrahepatic vena cava was ligated. Liver sinusoids were washed out through the portal cannula with 40 ml of Hanks solution. Leukocytes isolated from the wash-out fluid from the tumor bearing rats were named LALt (LAL for liver-associated-leukocytes; t for tumor) and those from normal rats LALn (n for normal). Leukocytes isolated from peripheral blood of with CC531 colon cancer metastases and normal rats were named PBLt and PBLn.

For identification of cellular antigens CD4, CD5, CD8, CD14, CD56, MHC class II, CD54, CD11a, CD11b and CD18, a direct immunofluorescence staining method was applied using specific mouse monoclonal antibodies. Cells were analyzed in flowcytometer FACStar.

Specimens of liver tissue were frozen in liquid nitrogen. Cryocut sections were stained with purified monoclonal antibodies, the same clones as for above described FACS analysis. Streptavidin-biotin-alkaline phosphatase complex technique was applied. Cell phenotypes were evaluated and their density in respective liver structures were scored from (^+^) to (^+++^).

## Results

### Phenotypes and adhesion molecules on sinusoidal wash-out LAL and PBL in normal rats

The sinusoidal wash-out LALn population contained 32.5 ł 4.0% of CD5^+ ^(T cells), 29.1 ł 6.1% of CD4^+^, 49.7 ł 5.1% of CD8^+^, 30.1 ł 16.3% of CD56^+^, 13.6 ł 3.6% of CD14^+^, and 16.7 ł 2.5% of MHC class II^+ ^cells. PBLn population was significantly different from LALn population in respect with CD5^+ ^(59.2 ł 2.8%), CD4^+ ^(50.6 ł 4.5%), CD8^+ ^(24.9 ł 3.0%) and CD56^+ ^(7.7 ł 3.5%) cell subsets (p &lt; 0.05).

In the LALn population, 72.0 ł 5.8% of CD11a^+^, 21.7 ł 4.6% of CD11b^+^, 58.5 ł 9.8% of CD18^+ ^and 29.0 ł 7.9% of CD54^+ ^cells were found. The PBLn values were lower than in LALn population for the CD11a^+^, CD18^+ ^and CD54^+ ^cells (p &lt; 0.05). They were 34.8 ł 4.9%, 29.1 ł 4.9% and 2.5 ł 2.2%, respectively.

### Phenotypes and adhesion molecules on sinusoidal wash-out LAL and PBL in liver CC531 tumor-bearing rats

The LALt population contained significantly more CD4^+ ^(40.3 ł 2.0%), CD14^+ ^(30.5 ł 11.4%) and MHC class II^+ ^(25.2 ł 2.6%) cells than the LAL1n population (p &lt; 0.05). The PBLn and PBLt populations did not differ in the distribution of leukocyte phenotypes.

The LALt contained significantly more CD11b^+ ^(42.57 ł 10.6%), CD18^+ ^(69.4 ł 7.4%) and CD54^+ ^(60.0 ł 16.8%) cells (p &lt; 0.05). No differences were found between PBLn and PBLt populations.

## Discussion

Around one million of leukocytes per gram of liver tissue can be washed-out from its sinusoids. This population contain more CD8^+ ^and CD56^+ ^and less CD5^+ ^and CD4^+ ^cells than peripheral or portal blood [1,7,8]. Data from our study remain in agreement with those published by others.

In the tumor-bearing livers the sinusoidal wash-out contained significantly more of CD14^+ ^and MHC class II cells than that of normal livers. It was due to liver intrinsic factors and not to the changes in blood leukocyte phenotypes. Accumulation of CD14^+^, DR^+ ^cells around tumor foci suggested the extraction of these subsets from the perfusing blood, possibly in response to the local chemotactic signals. Thomas et al and Griffini et al reported an increase in the number of KCs after intraportal injection of CC531 cells [9,10].

The leukocytes in the tumor tissue were CD54^+ ^and CD18^+^, some of them at the tumor-liver border expressed CD11a^+ ^and CD11b^+^. The expression of adhesion molecules on liver endothelial cells and liver leukocytes has been studied extensively [11-13]. The combinations of ICAM1-LFA1 and VCAM1-VLA4 were found to be most effective in normal and inflammatory conditions. Evidently more CD14^+ ^cells accumulating in the tumor vessels than in normal liver sinusoids suggests an additional mechanism of attraction of leukocytes to the growing tumor tissue. This notion is strengthened by findings of others that, in metastatic liver tumors, the endothelial cells only weakly express ICAM1 but no VAP-1 and VCAM1 [16].

Comparison of the populations of sinusoidal wash-out cells with those identified in and around liver tumor foci provided evidence for close similarities of both phenotypes of populations from these sources. This means these extracted from the circulation and tumor-adhering, cells reveal sore degree of specificity toward tumor antigens. The topographical location of the CD14^+ ^cells surrounding the tumor foci and accumulating in tumor blood vessels points to their role limiting tumor growth and expansion.

**Table 1:** Phenotypical analysis of cellular infiltrates in tumor bearing liver

Antigen	Cell infiltrates on the verge of the tumor	Perivascular infiltrates inside the tumor	Cells in tumor vessels	Cells in sinusoids of healthy liver
CD5	+/++	single	+	Single Lc
CD4	++	-/++	+	+ (KC,Lc) single
CD8	++	+/++	+	Lc
CD14	++/+++	+/+++	++	+ (KC,Lc) single
CD56	single	single	+/-	Lc
Class II	+/+++	++/+++	++	+ (KC, Lc)
CD54	+	+/++	+	-/+ (EC, KC, Lc)
CD18	++	+	+	+ (Lc)
CD11a	+	-	-	-
CD11b	+	-	-	+ (Lc)

Immunohistochemical identification and adhesion molecules on cells infiltrating liver CC531 tumor. Lc ł leukocytes, EC ł endothelial cells, KC ł Kupffer cells.
